# Living a Meaningful Life While Struggling with Mental Health: Challenging Aspects Regarding Personal Recovery Encountered in the Mental Health System

**DOI:** 10.3390/ijerph18052708

**Published:** 2021-03-08

**Authors:** Janne Brammer Damsgaard, Sanne Angel

**Affiliations:** Research Unit of Nursing and Healthcare, Institute of Public Health, Aarhus University, 8000 Aarhus, Denmark; angel@ph.au.dk

**Keywords:** mental health, recovery-orientation, psychosocial, personal recovery, social recovery, biopsychosocial, policy, education, personal formation

## Abstract

Based upon academic and clinical experience from Denmark, this article aims to highlight international research-based knowledge concerning challenging aspects about the understanding and implementation of recovery-oriented practice. Three key points are located: (a) An integrative biopsychosocial approach considering both the clinical and personal recovery perspectives is relevant for research and practice. (b) Barriers in implementing a recovery-oriented approach include both individual and systemic challenges. This is well documented in the research-based literature, highlighting the need for changes. (c) A shift from professional control to a service-user orientation is seen as crucial. Examples of a positive shift are seen, helping the health professionals in their development and practicing of skills and competences through education and personal formation. Within these perspectives, a paradigm shift from a one-dimensional biomedical approach to a biopsychosocial approach is suggested. Instead of focusing on rapid stabilisation and symptom relief as a clinical outcome, a humanistic approach building on social- and person-oriented values is fundamental for social and personal recovery leading to a meaningful life.

## 1. Introduction and History of Mental Health Services

The literature documents that people with mental illnesses can lead productive lives even while having symptoms and that many may recover. However, research shows that this demands a shift in focus that challenges both health professionals and organisations, and the public policy underpinning these.

Historically, the recovery movement initiated renewed hope for the individual struggling with mental illness. The recovery-oriented practice is in contrast to traditional perception and perspectives of professional practice and treatment within mental health care [1]. The aim towards a clinical recovery was well meant, but imposed a heavy burden on the individual. Full recovery has proven difficult to obtain, and within this clinical understanding, many have found it hard to create a meaningful life. Despite substantial research and efforts within a professional recovery-orientation in mental health services, users often do not experience that they are heard and receive the needed help [2,3,4,5,6,7,8,9,10,11]. This is a serious problem in our society where an increasing number of people are facing psychological difficulties. The WHO [12] estimates that in 2030, depression and loneliness will make up the largest international burden of diseases. 

Service users not being heard may derive from a history of being traditionally viewed as passive recipients of mental health care [13]. Despite being increasingly considered to have a legitimate voice in defining their own needs in recent years, there still remain challenges. Having gone from the role of patient to that of consumer, users have joined boards of provider agencies or developed resources in the form of peer support. However, if consumers are to have a defining voice in shaping the system to meet their needs, it seems timely for their input to be solicited, as well as on those issues that role change will influence for the future development and orientation of mental health policy. An example of a fundamental assumption that has shaped many mental health systems is the belief that individuals with serious disabilities lead better lives in the community than in the hospital. This assumption paved the way for the policy of de-institutionalisation, discharging severely disabled clients who either have been hospitalised continuously or have been unable to adapt to community living [13,14]. Despite considerable progress made in offering community-based services, there still remains work to be done, already pointed to in 1996 when Davidson and colleagues wrote their article “Hospital or community living? Examining consumer Perspectives in Deinstitutionalization” [13]. It is about time to begin to explore consumer input on mental health, on the debate and policy in general, and in regard to future re- or de-institutionalisation.

This article aims to highlight knowledge concerning challenging aspects about the understanding and implementation of a recovery-oriented practice. The aspects will be discussed within the view of: the shortcomings of clinical treatment; the shift from clinical recovery to personal recovery; what needs to be done to achieve personal recovery; what has been achieved in rendering care that is more recovery-oriented; what remains to be achieved in rendering care (challenges); and some ways by which this can be achieved.

## 2. The Shortcomings of Clinical Treatment

Evidence-based knowledge emphasises the importance of including other treatments and perspectives in regard to understanding mental health illnesses [15,16]. This understanding is supported by the fact that when addressing the inherent psychosocial aspects, half of all the people with serious psychosocial problems recover socially and one-fourth recover completely [17,18,19]. Based on a psychosocial approach, research confirms that recovery may unfold over time outside of formal treatment settings. For example, in contrast to the longstanding belief in inevitable deterioration in schizophrenia, older, but ground-breaking studies, found that up to 67 percent of people diagnosed with this condition experience significant improvements over time, and many fully recover [20,21,22,23,24,25,26]. In those people who did not fully recover, significant diversity was found in outcomes both within and across individuals [27]. Some people improved in certain areas (for example, employment) and not in others (such as symptoms), while the 33 percent who did not substantially improve could be characterised as being at various points on a broad spectrum, ranging from deterioration to clinical stability [28]. It is therefore understandable that the role of formal treatment in effecting these positive outcomes has been called into question. Questions about the role of treatment in recovery have been reinforced by studies suggesting that some people with long-term use of antipsychotic medications do less well over time than those who are not on long-term medications [29,30].

The psychosocial perspective has shown all-importantly to pave the way for participation in society, even when suffering from mental health challenges [11,31], especially because people can be assisted to overcome even the most serious mental illnesses if their case is understood from a personal development and lifeworld perspective instead of viewing them as chronic cases of disease [9,32]. This alternative professional view is based on existential and psychosocial rehabilitation, which includes a more societal responsibility in relation to citizens’ mental health and mutual effort to support recovery. 

## 3. The Shift from Clinical Recovery to Personal Recovery

The broad recognition of the ability of people with mental illnesses to participate in the mainstream of society has turned into a recovery movement. Among the confluence of factors is including longitudinal data, showing that many people eventually recover from serious mental illness [14]. The emergence and growth of the recovery movement has been supported by the revolution for self-government and autonomy of the 1960s. Thus, people “in recovery” have played an increasing role in advocating for person-centred care; greater self-determination for those with mental illnesses; and an enhanced focus on restoring functioning for individuals above and beyond symptom reduction [14]. 

Recovery is traditionally understood within the scientific community as sustained remission, or ideally as a disappearance of symptoms, accompanied by functional improvement (e.g., cognitive, social, and vocational functioning) and reduced use of medical health services [33]. This understanding equates recovery with returning to a state of full functioning, maybe due to compensation for the impairment. This has to do with sustained remission. It locates the concept within an illness frame of understanding, and equates recovery with long-term reduction or ideally, removal of symptomatology, accompanied by functional improvement. The key feature of this definition of recovery is that it is invariant across individuals. For example, Libermann and Kopelowicz [34,35] define recovery in schizophrenia as full symptom remission, full or part-time work or education, independent living without supervision by informal carers, and having friends with whom activities can be shared, all sustained for a period of two years. 

Another definition in regard to recovery has emerged, not from the mental health research literature, but from the increasingly coherent voices of individuals who have experienced mental illness and used mental health services. Within this patient perspective, early accounts of these experiences are shared in numerous narratives [36]. The narratives have pinpointed the fact that recognition of a meaningful life despite mental illness is crucial. This recognition has accentuated the individual and personal journey. Due to the emphasis on the individuality of recovery, the term “personal recovery” is used [37]. It is pointed out that recovery is indeed possible despite the presence of psychiatric symptoms. Coping with symptoms still constitutes a key feature, but recovery is regarded as more than that. Personal recovery refers to the individual process of adaptation and development through which the individual overcomes the negative personal and social consequences of mental disorder. This adaptation opens for a self-determined and meaningful life. It does not necessarily imply the return to life as before becoming ill or full functionality, but rather the growth beyond the premorbid sense of self [37]. This recovery growth has been measured as accepting mental illness; finding hope for the future; re-establishing a positive identity; developing meaning in life, taking control of one’s life through individual responsibility; spirituality, empowerment; overcoming stigma; and having supporting relationships [38].

## 4. What Needs to Be Done to Achieve Personal Recovery?

Personal recovery is founded on positive relational response. Therefore, knowledge of the process and adoption of a recovering attitude are necessary to deploy among professionals and at best, as a societal change. Unfolding the elements of the process, Leamy and Bird [39] conducted a systematic review of 97 studies. They identified five key elements: (i) connectedness, (ii) hope and optimism, (iii) identity, (iv) meaning, (v) purpose and empowerment. These elements became the basis for their development of the CHIME framework. Within this framework, the three recovery researchers have created a list with their Ten Top Tips for recovery-oriented practice [40,41]—see Box 1.

The thought-provoking element within these basic actions is that they solely address humanistic interactions with the service user. A human attitude is the foundation for health care professionals and health care services. Caring for the individual is, for example, the very reason for the nursing profession. In this context, when professionals educated to care are failing, it can be seen as a health care (and educational) system which does not manage to live up to its own ideals. Within this complex perspective, this can be seen as a system colonialising the lifeworld, causing both professional and user powerlessness. This is a fundamental area in need of focus and action.

Box 1 **Ten Top Tips for recovery-oriented practice.**

After each interaction, ask yourself did I

- Actively listen to help the person make sense of their mental health problems?
- Help the person identify and prioritise their personal goals for recovery—not my professional goals?
- Demonstrate a belief in the person’s existing strengths and resources in relation to the pursuit of these goals?
- Identify examples from my own ‘lived experience’, or that of other service users, which inspires and validates their hopes?
- Pay particular attention to the importance of goals which take the person out of the ‘sick role’ and enable them actively to contribute to the lives of others?
- Identify non-mental health resources—friends, contacts, organisations—relevant to the achievement of their goals?
- Encourage self-management of mental health problems (by providing information, reinforcing existing coping strategies, etc.)?
- Discuss what the person wants in terms of therapeutic interventions, e.g., psychological treatments, alternative therapies, joint crisis planning, etc., respecting their wishes wherever possible?
- Behave at all times so as to convey an attitude of respect for the person and a desire for an equal partnership in working together, indicating a willingness to ‘go the extra mile’?
- While accepting that the future is uncertain and setbacks will happen, continue to express support for the possibility of achieving these self-defined goals—maintaining hope and positive expectations?

## 5. What Has Been Achieved in Rendering Care That Is More Recovery-Oriented?

The literature advocates for the establishment of research activities that investigate and work towards fundamental changes within mental health care. An organisational and political focus is needed and has been seen internationally, for example, in Norway and the United Kingdom. Thus, there are experiences to be followed from both countries.

A reform of mental health care in Norway was initiated in 1998 with the establishment of the National Action Programme for Mental Health (NAPMH). This called for a major increase in the funding of mental health-related services, as well as a major reorganisation of these services [42]. NAPMH was an initiative to streamline and consolidate the fragmented services that were offered through various funding mechanisms, and to establish comprehensive, mental health care to local citizens. Five specific guidelines for the organisation of mental health services were established within NAPMH—see Box 2.

Box 2 **Five specific guidelines for the organisation of mental health services.**

- Preventive measures including early intervention and close monitoring
- Integration of mental health services with other health and social services
- Incorporation of users’ perspective, requiring user (and family) involvement and collaboration
- Voluntary treatment in an open and normal setting
- Promotion of continuing participation in a normal, ordinary life

The Norwegian programme has been an important initiative and has increased investigation for fundamental changes in the way mental health services are provided. Despite the impressive reform, implementation of the service models under this action programme have been met with barriers that are fundamentally rooted in the dominant culture of mental health care, including the resistance to shifting from the biomedical paradigm orientation to humanistic, person-oriented, and social-oriented mental health services and from professional control to service-user orientation [43]. According to Karlsson, “the process is going too slowly” and there is still a need for a public debate of the status within the Norwegian mental health system [3]. To Karlsson, despite good intentions, an internal and external closedness has been present, regarding the focus and discussions of fundamental challenges within the mental health system—for example, on the subjects: uncritical use of psychotropic drugs and too much use of coercive measures. 

The United Kingdom has succeeded in a shift toward emphasising commonality over differences in social services. Mental health policy has identified six outcomes to improve mental health: 1. Improving physical health, 2. Supporting recovery, 3. Improving experience of services, 4. Reducing avoidable harm, 5. Decreasing stigma, and 6. Improving the population’s well-being [44]. This policy shifted the balance away from a special policy for dealing with mental health problems and toward integration of mental health into mainstream social policy, a change reflected in the policy’s title—“No Health Without Mental Health”. According to Slade and Wallace [45], recovery has been embraced by professional groups. In England, for example, the principles of recovery have been adopted in clinical psychology [46], mental health nursing [47], occupational therapy [48], psychiatry [49] and social work [50]. However, there still remains work to be done.

## 6. What Remains to Be Achieved in Rendering Care That Is More Recovery Orientated?

The psychosocial approach has shown to be difficult to implement in a culture based on a traditional (biomedical) view of the individual. Irrespective of the documented effects of a recovery-oriented approach, studies and reviews indicate that successful implementation is limited and difficult to maintain [51,52,53,54,55,56,57,58,59]. It is emphasised that this difficult implementation is due to a proliferation of barriers at both the level of organisation and at the level of providers and users. The area is complex, including both systemic and individual challenges. Building on Anthony’s ideas, Slade [60] argues that the clinical framework underpinning most mental health services unfortunately locates problems “within” the individual. Furthermore, this often happens too late, when the suffering has been overlooked and becomes a problem affecting all aspects in a person’s everyday life. Clinical endeavours focus on the professionals changing people through treatment (therapy, skills training, etc.), so that they “fit in”, i.e., become “normal” and “independent” of support and services [60]. 

The aspects are complex and are not a question of either clinical or personal recovery. An integration of the biomedical/clinical and the psychosocial framework—a biopsychosocial approach—is relevant. The dynamic biopsychosocial model was introduced as early as 1980 by Engel [61], elaborating human health as a product of the reciprocal influences of biological, psychological, interpersonal, and macrosystem contextual dynamics that unfold over personal and historical time. The importance of these influences varies within a person over time; hence, dynamic interpersonal, biological, and psychological systems interact with contextual factors to shape health over the life span [62]. Within this perspective, recovery is no longer seen as an event occurring solely within an individual, which is implicitly assumed by the concept of clinical recovery (e.g., symptom reduction). Recovery is a dynamic interplay between the individual and its environment. Consequently, the construction of recovery instruments becomes more complex. This suggests that an integrative biopsychosocial approach considering both the more objective and the more subjective recovery perspectives could be promising for research and practice [33,62,63].

Seen within this perspective, the wish for “getting better” or ceasing to need support is acknowledged. Recovery (clinically and personal) comes to mean recovering a life—about the right to participate in all facets of civic and economic life as an equal citizen [64]. Hence, participation and inclusion do not involve changing people to fit in, but changing the (health care) system and society to be inclusive. To Slade [65], the challenges exist in the form of (unintended) barriers in the environment that prevent people from living. This can, for example, be standardising health services or employment services inducing structures and systems [9,14,65,66,67]. Within this context, a change can only happen by confronting and challenging health care systems to reduce barriers that impede and thwart people’s efforts to live independently and gain control over their lives and the resources needed. 

A literature review by Madsen shows that many mental health care organisations fail when adapting inclusive principles and work recovery-oriented [68]. This is supported by Madsen’s research [2]. It appears that the health professionals at the psychiatric wards do have *knowledge* about recovery and a recovery-oriented approach, and that they do have *intentions* about integrating this in their daily work. Even so, health professionals have *difficulties with establishing consensus* about what to understand by recovery and to *put their knowledge to work in practice*. According to Madsen, neither patients nor health professionals find that a recovery-oriented approach is an integrated part of the clinical practice, and they only see a *limited collaboration between patients and health professionals*. A medical/medicinal and crisis-driven practice is dominant in psychiatric wards. Challenges with working in a recovery-oriented way are assigned to the *absence of a clear ideology* in daily work. Both the *absence of a clear recovery ideology and practice* are assigned to *limited resources and capacity*, as well as *counterproductive structures and procedures in the organisation* of the wards [2]. Similar results are found within the study of Jørgensen et al., focusing on intersectoral care in mental health [69]. The results emphasise the need of an organisational as well as an educational focus in mental health services [41,70]. 

## 7. How Can It Be Achieved?

In the article “Where are we now? Recovery and Mental Health”, research shows that it is a complex area to evaluate. Among the results of Slade’s review [45], the following are pinpointed—see Box 3.

Box 3 **“Where are we now? Recovery and Mental Health” (Slade 2017).**

- Empirical research and interventions which improve connectedness are limited.
- A systematic review of interventions for fostering hope identify promising interventions including collaborative illness management strategies, fostering positive relationships, peer support and support for setting and attaining realistic personally valued goals.
- Receiving peer support from peer support workers or mentors who have themselves experienced mental health difficulties has been shown to increase hopefulness when compared with treatment as usual, with additional recovery benefits noted for the peer workers themselves.
- Interventions to support the development and maintenance of a positive identity are lacking. Approaches which are worth developing as intervention technologies include life-story work, “Tree of Life” and narrative therapy.
- Meaning and purpose in life find expression in many ways, but one key aspect is through spirituality and religion. Unfortunately, these domains are not only deprioritised but often actively excluded from clinical discourse.
- Several interventions have been developed which target personal responsibility and control, including advance directives, joint crisis plans, and shared decision making. Evaluations have included randomised controlled trials and quasi-experimental designs, and have shown a range of positive outcomes including reduced hospitalisation, increased social support, goal setting, and goal attainment.

Standing on international research and at the intentions and experiences from Norway and the United Kingdom, it is clear that understanding and integration of person-oriented and social-oriented shifting from professional control to service-user orientation is crucial. Within this focus, an overall implementation strategy is needed paradigmatically, politically, and organisationally to support the health professionals in their development and practicing of skills from education to personal formation, by supporting them in maintaining, preserving, and furthering their professional focus and involvement [19]. Patient organisations still play an important role in drawing attention to this need for change. Thus, this is a focal point in the recommendations of the organisation—Danish Society of Psychosocial Rehabilitation—which in *Ten recommendations to the Danish politicians* [71] points out that the curriculum that trains staff for the regional psychiatric system is by and large based on biomedical knowledge, which also to a large extent goes for the social care element [71]. They argue for the introduction of pluralism in the curriculum taught as well as for competences with a focus on awareness that “the relational aspect always comes before the methodology” [71]. It is a question about encouraging and developing the ability of the employees to hope, be creative, caring for and showing compassion, be realistic and to develop resistance based on life stories and individual reasons for psychological challenges. What is required is developing an awareness to our lifeworld, which leads to the formative education of competent and wise human beings who are in possession of sensitivity and understanding [19,72,73]. The personal qualities of health professionals are important to develop based on experiences in clinical settings that determine both what sensitivities and respective insensitivities we will have access to in dealing with the potential meanings we encounter. At its heart, the educational process consists of learning how we relate to or (have to) position ourselves in relation to the various spheres of action and life [19,74,75]. From this perspective, the relation can only be established when we discover that we can achieve or move something, i.e., that the experience “responds” to us within our lifeworld.

## 8. Key Points

The challenges in regard to (personal) recovery are complex and require a nuanced perspective. The following three key points are worth paying attention to:

First, recovery is a question of neither biomedical/clinical nor psychosocial recovery—it is an integration of the clinical and the psychosocial framework. Therefore, a biopsychosocial approach is relevant. Within this perspective, recovery is no longer seen as an event occurring solely within an individual, which is implicitly assumed by the concept of clinical recovery (e.g., symptom reduction). Here, recovery becomes a dynamic interplay between the individual and its environment. This suggests that an integrative biopsychosocial approach considering both the more objective and the more subjective recovery perspectives could be promising for research and practice.

Second, there are barriers in implementing a recovery-oriented approach at both the level of organisation and at the level of providers and users. The area is complex, including both individual and systemic challenges. It is found that health professionals in psychiatric services do have knowledge about recovery and a recovery-oriented approach, and they do have intentions about integrating this in their daily work. Even so, health professionals have difficulties with establishing consensus about what to understand by recovery and to put their knowledge to work in practice. This can be seen as a health care (and educational) system which fails to live up to its own ideals of humanistic actions—the system colonialising the lifeworld, causing both professional and user powerlessness. This calls upon a policy shifting the balance away from a special policy for dealing with mental health problems and toward integration of mental health into mainstream social policy.

Third, based upon international research and experiences from both Norway and the United Kingdom, it is clear that shifting from a professional control to service-user orientation is crucial. Within this focus, allowance must be made for a shift in culture, helping the health professionals in their development and practicing of skills and competences from education to personal formation, by supporting them in maintaining, preserving, and furthering their professional focus and involvement. It is central to encourage and develop the ability to hope, be creative, caring for and showing compassion, with the intentions of creating conditions for living meaningful lives while struggling with mental health challenges.

## 9. Conclusions

It is found that a recovery-oriented practice added to treatment improves the process of striving for a life worth living. A substantial societal effort is needed. Within this perspective a paradigm shift from a one-dimensional medical approach to a biopsychosocial approach is suggested. Humanistic values are crucial as a basis for the health professionals’ work and education. Instead of focusing on rapid stabilisation and symptom relief as a clinical outcome, this humanistic focus enables personal recovery, leading to a meaningful life. Therefore, an approach building on social-oriented and person-oriented values is the way forward.

## Data Availability

No new data were created or analyzed in this study. Data sharing is not applicable to this article.
